# Predictors of change of health workers’ knowledge and skills after the Helping Mothers Survive Bleeding after Birth (HMS BAB) in-facility training in Tanzania

**DOI:** 10.1371/journal.pone.0232983

**Published:** 2020-05-18

**Authors:** Fadhlun Alwy Al-beity, Andrea Barnabas Pembe, Gaetano Marrone, Ulrika Baker, Claudia Hanson

**Affiliations:** 1 Department of Global Public Health, Karolinska Institutet, Stockholm, Sweden; 2 Department of Obstetrics and Gynaecology, Muhimbili University of Health and Allied Sciences, Dar es salaam, Tanzania; 3 Department of Disease Control, London School of Hygiene and Tropical Medicine, London, England, United Kingdom; Liverpool School of Tropical Medicine, UNITED KINGDOM

## Abstract

**Background:**

Our study aimed to assess the effect of *Helping Mothers Survive Bleeding after Birth* on knowledge and skills of health workers and whether such effect varies by health workers characteristics.

**Methods:**

Nested in a cluster-randomised trial to assess the effect of the training on health outcomes, we assessed changes in knowledge and simulated skills in 61 facilities. The assessments were done i) before, ii) immediately-after training session and iii) at 10-month follow-up for subset of health-workers of implementation facilities as defined by the trial. We used a self-administered questionnaire and Objective Structures Clinical Examinations to assess three skill sets: Active Management of Third Stage of Labour, removal of retained placenta and management of severe postpartum haemorrhage. We computed summary statistics and used the paired t-test to assess change of knowledge and skills immediately post-training and at 10-month follow-up. Linear regression was done to assess association of scores and health worker characteristics.

**Results:**

Of the 636 health workers included, 606 (96.7%) and 591 (91.4%) completed the knowledge and skills assessments, respectively. Majority of the participants (68%) were nurse-midwives. Knowledge scores increased by 15 percentage-points from 77.5% to 93% (95% CI 14.3, 16.3, p-value <0.000), and skills scores by 47 percentage-points (95% CI 46.5, 49.2, p-value <0.000) from 37.5% to 83%. There was a 4.0% decline of skills at 10-month follow-up. The decline was higher in auxiliary staff (-11.8%) and least in nurse-midwives (-2.1%) p-value <0.001. Health workers who assisted less than 5 deliveries in the last month, those who never attended postpartum haemorrhage in-service training and profession experience >8 years were associated with lower mean skill change immediately post-training.

**Conclusion:**

Our study supports the potential of the *Helping Mothers Survive Bleeding after Birth* training to increase knowledge and skills of postpartum haemorrhage among all professional groups. Auxiliary staff benefited most from the training but also showed higher skill decline at 10-month. Our study highlights the importance to disaggregate knowledge and skills by health workers characteristics.

## Background

To end preventable deaths and reduce the maternal mortality ratio (MMR) below 70 per 100,000 livebirths by 2030, countries need to invest in strengthening the health workforce [1]. Common health workforce challenges include critical absolute shortages in availability, high rates of attrition, unequal distribution, inadequate skills and unmotivated staff [1–4]. These problems are among other things rooted in poor pre-service training that is often rather theoretical and classroom based, than practice-oriented, focusing on numbers instead of quality education. Moreover, trainings often fail to promote inter-professional collaboration for childbirth care [3].

In-service training is a frequent method to address the lack of skills among the existing health workforce in order to strengthening knowledge, skills and attitudes [5], commonly used in maternal and newborn care to improve health workers performance and health outcomes [6, 7]. Evidence suggesting results are always better with multifaceted interventions rather than single interventions and training interventions with structured follow-up interventions are encouraged [7–9].

While various trainings may address similar content in terms of intrapartum and newborn care, they differ in content details, duration, site of implementation, training modalities, use of simulation and other components such as teamwork [9]. A recent review of trainings for maternal and newborn health summarised that knowledge, skills and confidence of health workers following such training generally improve [10–12]. Clinical practices are also reported to improve whereas studies that report health outcomes vary some showing improvements [13–17] and others report no effect following such training [18].

However, few studies have assessed determinants of knowledge and skills improvement such as factors associated with knowledge gains, knowledge retention. We only found one 9-country study that reported on associations of knowledge and skill acquisition following emergency obstetric training and this put forward associations with country, health workers’ profession and years of professional experience but no association with proportion of maternity work done [12]. Two reviews found a decline of knowledge and skills 6–12 months after training, but the decline did not reach the levels found before the training [19, 20].

The *Helping Mothers Survive Bleeding after Birth (HMS BAB)* training is a competency-based simulation training delivered in health facilities to all health workers in a maternity unit [21]. Implemented and evaluated in several low and middle income-countries, the HMS BAB training is reported to be acceptable [22, 23] and leads to improved knowledge and skills scores [24]. The improved knowledge and skills are retained at 6–12 months post training [14]. Also, improved clinical outcomes have been described [13]. There is, however, limited evidence if certain health worker characteristics such as professional experience and level, such as cadres, years of professional experience, number of deliveries assisted in the last month or attending prior in-service training are related to knowledge and skills change. Knowing such differences will be important to target re-training and better allocation of limited resources.

As other low- and middle-income countries, Tanzania has high maternal mortality of 556 deaths per 100,000 live birth, 25% of which are contributed by haemorrhage [24, 25]. Low skills and implementation rates of PPH prevention interventions are among challenges to reducing PPH deaths and morbidities. Observational studies in the country report as low as 29% of women received Active Management of Third Stage of Labour (AMTSL) within one-minute of childbirth [26]. Only 69% of health workers working in health centres self-report to perform AMTSL [27].

Nested in a cluster-randomised trial to assess the effect of the training on health outcomes, we assessed changes in knowledge and simulated skills before and after the training in 61 facilities we assessed whether the effect of the HMS BAB training on change in knowledge and skills, including retention of the same 10-month follow-up, varied by health workers’ characteristics [13, 28]

## Methods

This before-after study with no comparison group was done within the HMS BAB cluster trial setting registered with Pan African Clinical Trials Registry, PACTR201604001582128 on 12 April 2016, details published elsewhere [28]. Sixty-one health facilities were included, 23 were district hospitals and 38 were large health centres. All were rural or semi-rural health facilities; most were government own (84%) and had similar characteristics such as number of clinicians, nurse-midwives and availability of oxytocin as published in the main HMS BAB trial [13, 28]

Health workers in maternity ward include medical doctors, other clinicians who are either clinical officers with three years of training or Assistant Medical officers who have additional skill training of two years [29]. All nursing cadres were included in nurse-midwives, including registered nurse-midwives with diploma, advanced diploma and degree or enrolled nurse midwives with certificates [30]. Auxilliary staff included were medical assistants.

### The HMS BAB training

The HMS BAB training intervention was provided to all maternity health workers using the 2013 basic HMS BAB curriculum developed by Laerdal Global Health and Jhpiego [21, 22] as part of our HMS BAB trial [13, 28].

First Jhpiego HMS BAB master trainers trained 12 district-HMS BAB trainers during a one-week training in a central location and later travelled to the districts where the HMS BAB in-facility trainings were conducted [13, 28]. One-day-long in-facility training were provided to all available health workers in maternity units, following same schedule and content. In each facility, district trainers selected two health workers using a specific criterion to become “peer practice facilitators”. These peer practice facilitators were further trained to lead eight-weekly so called “practice drills” that were mandatory sessions within their facilities. These “practice drills” were based on clinical scenarios and were to be conducted for 30–40 minutes per week. This training technique is referred to as “low dose high frequency” as learners are exposed to frequent repetitive learning exercises. Health workers who performed poorly during the sessions were given more practice until they mastered the specific simulated skill. Participants received refreshments during the in-facility training sessions. All facilities received Mama Natalie training kits for use during these sessions.

### Participants

In this secondary data analysis, we included all the trained maternity ward health workers of participating health facilities: 636 health workers were trained. 337 in January 2016 in 31 intervention facilities of HMS BAB trial and 299 in January 2017 in 30 comparison facilities after the end of the trial. The 10-month assessment in a subset of health workers were done only in intervention facilities for operational reasons.

### Assessments and tools

We used the standard Jhpiego pre- and post-training assessments [21, 22] which comprised a written knowledge test with 15 points multiple choice and True/False questions. The tests were made available in the local (Swahili) language and were self-administered. The written test was followed by simulation session where each participant was asked to manage a simulated case of PPH including recognition of retained placenta practice of simulated skills to prevent, detect and do basic management of PPH. Each theoretical session was followed by a specific pre-training assessment, demonstration followed by discussions and later post-training assessment of the skill. Skill assessments were done by validated Objective Structured Clinical Examinations (OSCEs) checklists using three simulate scenarios: performing Active Management of Third Stage of Labour (AMTSL), managing retained placenta and managing severe PPH secondary to uterine atony [22, 23]. For a subset of health workers re-assessment using same tools was performed at 10-month follow-up.

Additionally, the participants’ professional qualification, years of professional experience working in the maternity ward, number of deliveries assisted in the last 30 days, and whether they had ever received previous in-service training in AMTSL or PPH was documented.

### Data handling and analysis

Data were manually entered in MS excel and checked for completeness. To analyse the data, we created a new score variable combining the three OSCE skill scores into a mean skill score. Analysis was done by STATA software version 15. Tables of summary statistics were prepared to describe the data: mean or median and standard deviation or Interquartile Range (IQR) were used to summarize continuous variable, frequencies and percentages to summarize categorical variables. Years of professional experience was categorized into <2 year, 2–4 years, 5–7 years, and ≥ 8 years. Number of assisted deliveries in the last one-month was categorized in ≤5 deliveries, 6–15 deliveries, and ≥16 deliveries assisted in the last one-month. Professional qualifications were categorized into (1) medical doctors (2) other clinicians (assistant medical officers and clinical officers); (3) nurse-midwives included all nurse-midwives with diploma and above; (4) auxiliary staff and (5) others included laboratory technicians. The facility level was categorized into hospitals, larger health centres that performed Caesarean sections (C-sections) and lower health centre without C-sections. Previous in-service training (ever) of AMTSL or PPH was set as a binary variable.

The outcome variables were absolute scores and mean change of scores in knowledge and aggregate OSCE skills. Aggregate OSCE skill score was an average of all three OSCE scores: AMTSL, simulated retained placenta and severe PPH due to atony and is referred as skill scores throughout. Immediate change of scores was calculated as the difference between immediate post-training score and pre-training score and change at follow-up (decline) was calculated as difference between scores at follow-up and that of immediate post-training All scores were summarized and mean with 95% confidence intervals presented in tables. We compared individual health workers’ knowledge and skill scores to assess change immediately and that at follow-up period using paired t-test and reported significance results at p-value <0.05.

We used linear regression analysis to assess the predictors of change of knowledge and skill scores 1) immediately after the training and 2) at 10-month follow-up, for each of the independent variables and present coefficients with 95% confidence intervals. Significance level was set at p<0.05.

### Ethics and consent

Ethical clearance was granted from Muhimbili University of Health and Allied Sciences IRB with permit number 2015-11-18/AEC/Vol X/71 and 2016-12-14/AEC/Vol XIII/03. All participants were informed about the study and written informed consent sought and obtained. The benefit of participating in the study was improving knowledge and skills that lead to better performance and job satisfaction. There was no potential harm from participating in the training, except for the inconvenience of assessments. All participants were given codes to ensure follow up of scores without identifying individual performance. All personal identifiers were removed from electronic data.

## Results

**Fig 1:**
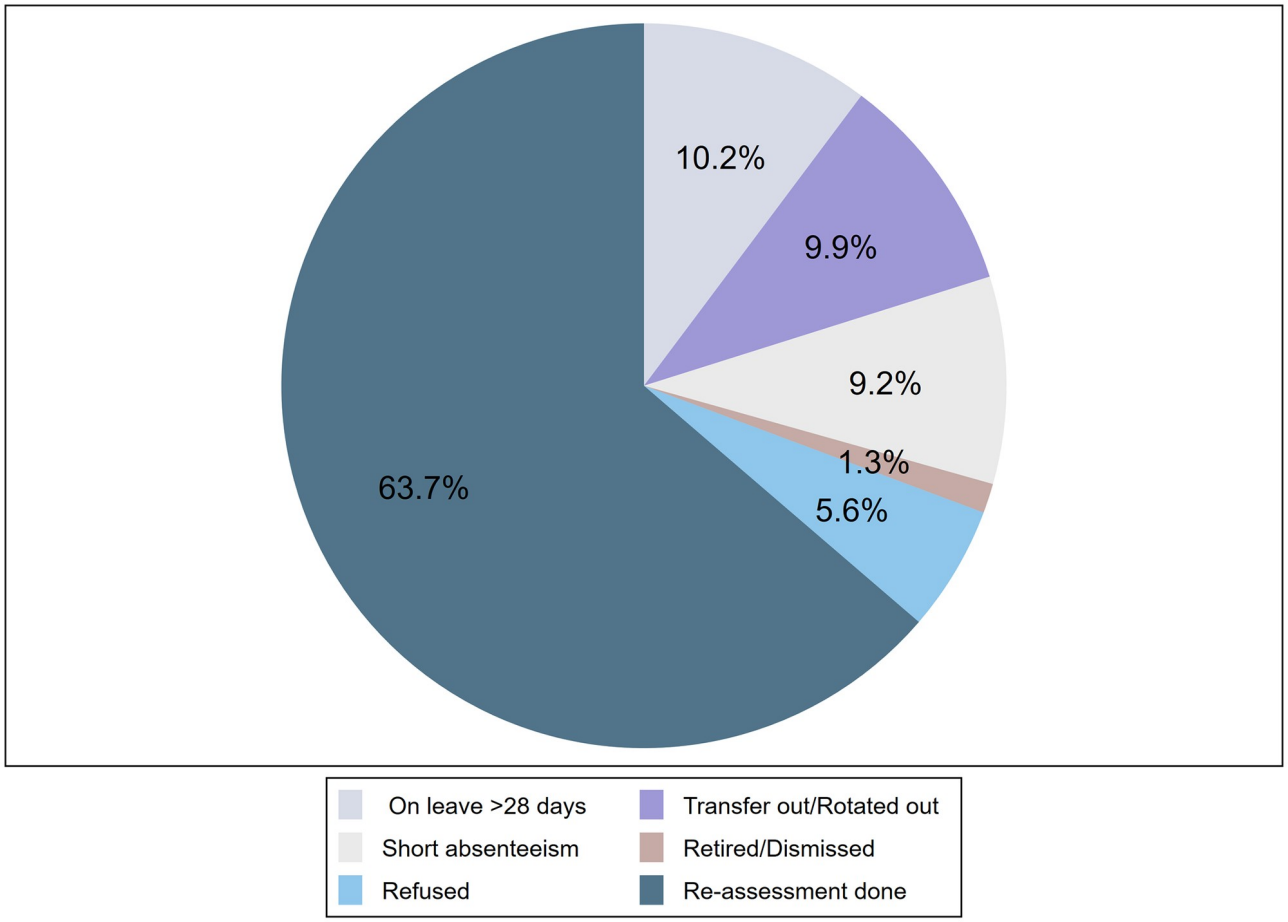
Availability of health workers within the facilities at 10-month follow-up (n = 303). A group pf 303 health workers were followed-up at 10-month after training, 63.7% (193/303), were available for re-assessment. The remaining 110 health workers were unavailable: 10.2% (31/303) were on annual, maternity or sick leave, 9.9% (30/303) were transferred/rotated out of maternity ward, 9.2% (28/303) were on short absenteeism attending training outside of facility, 5.6% (17/303) refused to be re-assessed and 1.3% (4/303) had left their profession: retired or dismissed.

A total of 636 health workers received the training, 95.2% (606/636) completed the knowledge assessment and 92.9% (591/636) completed the knowledge and skills for both pre- and immediate post-training assessment. Twenty-five participants, 3.9% (25/636) dropped out of the training; thus, only the pre-training knowledge assessment is available for these participants (S1 Fig). A sub-set of 303 participants who were included in January 2016 in the training were traced for reassessment at 10-month. Only 193 (63.7%) were available at this time. A total of 110 participants were absent for various reasons (Fig 1).

A total of 636 health workers participated in the study, 435 (68.4%) nurse-midwives, 56 other clinicians (8.8%), 13 medical doctors (2%) and 128 (20.1%) auxiliary staff (medical attendant), 4 (0.6%) health workers were missing their profession level as shown in Table 1. There was no medical specialist. The participating health workers assisted a median of 2 (Interquartile range (IQR) 2–10) of deliveries in the last month. The median number of years of professional experience working in the maternity ward was 3 (IQR 1,8) years. Half of the health workers (52.5%) assisted less than five deliveries in the last month. (Table 1). Only 46% and 41% of health workers had ever received AMTSL or PPH in-service training.

**Table 1:** HMS BAB trained health workers by professional characteristics (n = 636).

Health worker characteristic	Medical Doctors	Other Clinicians	Nurse-midwives	Auxiliary staff	Missing	All
	*n (%)*	*n (%)*	*n (%)*	*n (%)*	*n (%)*	*n (%)*
***Profession***	13 (2.0)	56 (8.8)	435 (68.4)	128 (20.1)	4 (0.6)	636 (100)
***Number of deliveries assisted in the last month***						
*≤5 del/month* *	10 (77.0)	43 (76.8)	193 (44.4)	86 (67.2)	2 (50.0)	334 (52.6)
*6–15 del/month*	2 (15.4)	9 (16.1)	167 (38.4)	35 (27.3)	2 (50.0)	215 (33.7)
*≥16 del/month*	1 (7.7)	4 (7.1)	75 (17.2)	7 (5.4)	0	87 (13.7)
*Median number of deliveries [IQR]*	3	2	7	4		5
	[0–5]	[0–5]	[3–13]	[2–8]		[2–10]
***Years of professional experience***						
*< 2 years*	5 (38.5)	9 (16.1)	111 (25.5)	42 (32.8)	2 (50.0)	169 (26.6)
*2–4 years*	6 (38.5)	13 (23.1)	150 (34.5)	29 (22.7)	1 (25.0)	198 (31.1)
*5–7 years*	0	5 (8.9)	53 (12.2)	13 (10.2)	0	71 (11.2)
*≥ 8 years*	2 (15.4)	21 (37.5)	97 (22.3)	37 (28.9)	0	157 (24.7)
*Missing*	1 (7.7)	8 (14.3)	24 (5.5)	7 (5.5)	1 (25.0)	41 (6.5)
*Median years of professional experience [IQR]*	2	5	3	3		3
	[0–3.5]	[2–14]	[1–7]	[1–10]		[1–8]
***Facility level***						
*Hospital*	12 (92.3)	24 (42.8)	251 (57.7)	49 (38.3)	1 (25.0)	337 (53.0)
*Health centres performing C- sections*	1 (7.6)	11 (19.6)	48 (11.0)	12 (9.4)	1 (25.0)	73 (11.5)
*Health centre*	0	21 (37.5)	136 (31.3)	67 (52.3)	2 (50.0)	226 (35.5)
***Ever attended prior in-service AMTSL training***						
*Yes*	6 (46.2)	33 (58.9)	224 (51.5)	33 (25.8)	1 (25.0)	296 (46.7)
*No*	7 (53.9)	19 (33.9)	195 (44.8)	89 (69.5)	2 (50.0)	310 (49.1)
*Missing*	0	4 (7.1)	16 (3.4)	6 (4.7)	1 (25.0)	27 (4.3)
***Ever attended prior in-service PPH training***						
*Yes*	4 (30.7)	30 (53.6)	212 (48.7)	15 (11.7)	0	261 (41.0)
*No*	*8 (61*.*5)*	*18 (32*.*1)*	*200 (46*.*0)*	*103 (80*.*5)*	2 (50.0)	*331 (52*.*0)*
*Missing*	*1 (7*.*7)*	*8 (14*.*3)*	*23 (5*.*3)*	*10 (7*.*8)*	2 (50.0)	*44 (6*.*9)*

*Of those with ≤ 5 deliveries in the last one-month, 126 did not assist in-any delivery and 108 assisted between 1 and 5 deliveries.

Main outcomes for pre-training, immediate post-training and 10-month follow-up scores for knowledge and aggregate skill scores by health workers’ characteristics are shown in Table 2. We observed an overall knowledge score of 74.2% (95% CI 72.8–75.5%) at pre-training, a knowledge score of 89.2% (95% CI 87.5, 90.9) immediately after the training and 85.4% (95% CI 83.5–87.3%) at the 10-month follow-up. The medical doctors had higher pre-training knowledge scores at 90.2% (95% CI 85.2,95.2) than other professions and increased to 95.4% (95% CI 91.8,98.9). Knowledge score for other clinicians increased from 75.7% (95% CI 71.6, 79.8) to 89.9% (95% CI 87.1, 92.7) and nurse-midwives score from 77.4% (95% CI 76.0,78.8) to 92.1% (95% CI 91.1,93.1). The auxiliary staff had the lowest pre-training knowledge score 60.9% (95% CI 58.1, 63.8) that increased to 80.3% (95% CI 77.4–83.1%). Overall skill scores increased immediately from 38.3% (95% CI 36.8, 39.6) to 85.4% (95% CI 84.3, 86.5) immediately post-training. Knowledge and skill score increase was statistically significant across all professions (p-value <0.001). Pre-training and immediate post-training knowledge and skill scores did not differ by number of deliveries assisted in the last month, years of profession experience and facility level.

**Table 2:** Knowledge and skills scores pre-, immediately after and at 10-month follow-up of the HMS BAB training by health worker’s characteristics.

	*Knowledge scores*						*Skill score*					
	Knowledge Pre-training		Knowledge Immediately post		Knowledge 10-month follow-up		Skills Pre-training		Skills Immediately post		Skills 10-month follow-up	
*Health worker characteristic*	*n*	Mean score	n	Mean score	n	Mean score	n	Mean score	n	Mean score	n	Mean score
		(95% CI)		(95% CI)		(95% CI)		(95% CI)		(95% CI)		(95% CI)
*All*	*633*	74.2 (72.8–75.5)	605	89.2 (87.5, 90.9) ***	192	85.4 (83.5–87.3) *	629	38.2 (36.8, 39.6)	592	85.4 (84.3, 86.5) ***	191	80.8 (77.8, 82.4) **
***Profession***												
*Medical doctors*	*13*	90.2 (85.2,95.2)	11	95.4 (91.8,98.9) *	2	93	13	49.1 (39.9.58.2)	11	87.0 (79.9, 94.2) ***	2	80 (64.9, 95.1)
*Other clinicians*	*54*	75.7 (71.6, 79.8)	52	89.9 (87.1, 92.7) ***	18	84.6 (77.5, 91.6)	55	38.0 (33.3.42.8)	53	83.9 (79.8, 87.9) ***	18	78.3 (71.4, 85.2)
*Nurse-midwives*	*434*	77.4 (76.0,78.8)	416	92.1 (91.1,93.1) ***	143	86.2 (84.0, 88.5) **	432	41.0 (39.3, 42.6)	404	88.2 (87.1, 89.3) ***	143	83.4 (81.4, 85.4)
*Auxiliary staff*	*128*	60.9 (58.1, 63.8)	123	80.3 (77.4, 83.1) ***	29	75.4 (69.3, 81.5)	125	27.8 (25.2, 30.4)	120	76.6 (73.9, 79.3) ***	27	63.8 (54.7, 72.9) *
***Number of deliveries assisted in the last month***												
*≤ 5 del/month*	*333*	73.2 (71.3, 75.1)	316	88.7 (87.2, 90.1) ***	97	82.7 (79.5, 85.8) **	329	36.5 (34.5, 38.4)	311	83.3 (81.7, 85.0) ***	95	76.2 (72.7, 79.7) **
*6–15 del/month*	*214*	75.5 (73.4, 77.7)	206	91.0 (89.5, 92.5) ***	64	85.4 (82.2,88.7) **	214	39.6 (37.2, 42.1)	203	87.9 (86.2, 89.3) ***	64	84.3 (81.1, 87.5)
*≥16 del/month*	*86*	74.5 (71.1, 77.9)	84	88.8 (86.0,91.5) ***	32	87.7 (83.1,92.3)	86	41.0 (37.4, 44.5)	78	87.2 (84.7, 89.4) ***	32	83.2 (78.4, 88.1)
***Years of professional experience***												
*< 2 years*	*167*	73.4 (70.8,76.0)	161	89.5 (87.5, 91.4) ***	53	82.7 (78.5, 87.8)	166	35.9 (33.2, 38.6)	157	85.4 (83.4,87.4) ***	52	79.1 (74.8, 83.5)
*2–4 years*	*197*	75.8 (73.6,78.1)	185	90.1 (88.4, 91.9) ***	54	84.1 (80.1,88.0) *	196	40.4(38.0, 42.9)	180	88.6 (87.1, 90.2) ***	54	81.7 (78.2, 85.2) *
*5–7 years*	*71*	76.0 (72.1, 80.1)	71	89.8 (86.9, 92.7) ***	24	85.7 (79.1,92.3)	71	39.7 (35.1, 44.3)	70	85.3 (82.1, 88.5) ***	23	75.0 (65.0, 85.0) *
*≥ 8 years*	*157*	73.3 (70.6,75.9)	154	89.3 (87.2, 91.2) ***	44	86.8 (83.9, 89.7)	155	37.1 (34.4, 39.8)	151	83.5 (81.3, 85.7) ***	44	79.5 (75.2, 84.3) *
***Facility level***												
*Hospital*	*336*	75.6 (73.8,77.3)	316	90.0 (89.6, 92.2) ***	98	83.6 (80.5, 86.8) **	331	37.5 (35.7, 39.5)	302	84.9 (83.4, 86.4) ***	98	79.4 (76.4, 82.3)
*Health centres performing C- sections*	*72*	71.8 (68.2, 75.4)	67	85.4 (82.2, 88.5) ***	34	87.2 (83.1, 91.5)	73	37.5 (33.3, 41.7)	66	85.3 (82.4, 88.2) ***	33	82.8 (78.9, 86.1)
*Health centre*	*225*	72.9 (70.5, 75.3)	223	88.7 (86.9,90.5) ***	61	84.1 (80.7, 87.4) **	225	39.3 (36.9, 41.6)	224	86.1 (84.2, 87.9) ***	60	79.8 (74.8, 84.7) **
***Ever attended prior in-service AMTSL training***												
*Yes*	*296*	76.7 (74.8, 78.5)	290	90.6 (89.2, 92.0) ***	78	86.3(83.6, 89.1) *	294	38.8 (3.8, 40.9)	283	86.2 (84.8, 87.6) ***	78	82.3 (79.3, 85.2) *
*No*	*310*	72.4 (70.5,74.3)	293	88.3 (86.9,89.8) ***	102	83.8 (80.6, 86.9) *	308	37.1 (35.1, 39.0)	287	84.6 (82.9, 86.3) ***	100	77.8 (74.2, 81.3) *
***Ever attended prior in-service PPH training***												
*Yes*	*261*	78.7 (76.8,80.5)	255	91.7 (90.3, 93.2) ***	72	85.9 (82.7, 89.0) *	259	40.8 (38.8,42.9)	250	88.2 (86,8, 89.5) ***	72	83.1 (80.4, 85.8) **
*No*	*328*	71.0 (69.1, 72.9)	312	87.7 (86.3, 89.2) ***	101	84.6 (81.5, 87.6) *	326	36.0 (34.0, 37,9)	304	83.5(81.9, 85.2) ***	99	78.1 (74.7,81.5) *

*p-value <0.05,

**p-value <0.01

***p-value <0.001

Paired t-test was used to compare test scores immediately post training and that of pre-training scores: paired t-test were available for 605 health workers with immediately-post/pre-training scores for knowledge, and 591 health workers with skill scores. Paired t-test between scores at 10-month follow-up and scores immediately post-training were done with health workers having both 10-month follow-up/immediately-post training scores 183 knowledge scores and 172 skill scores.

**Fig 2:**
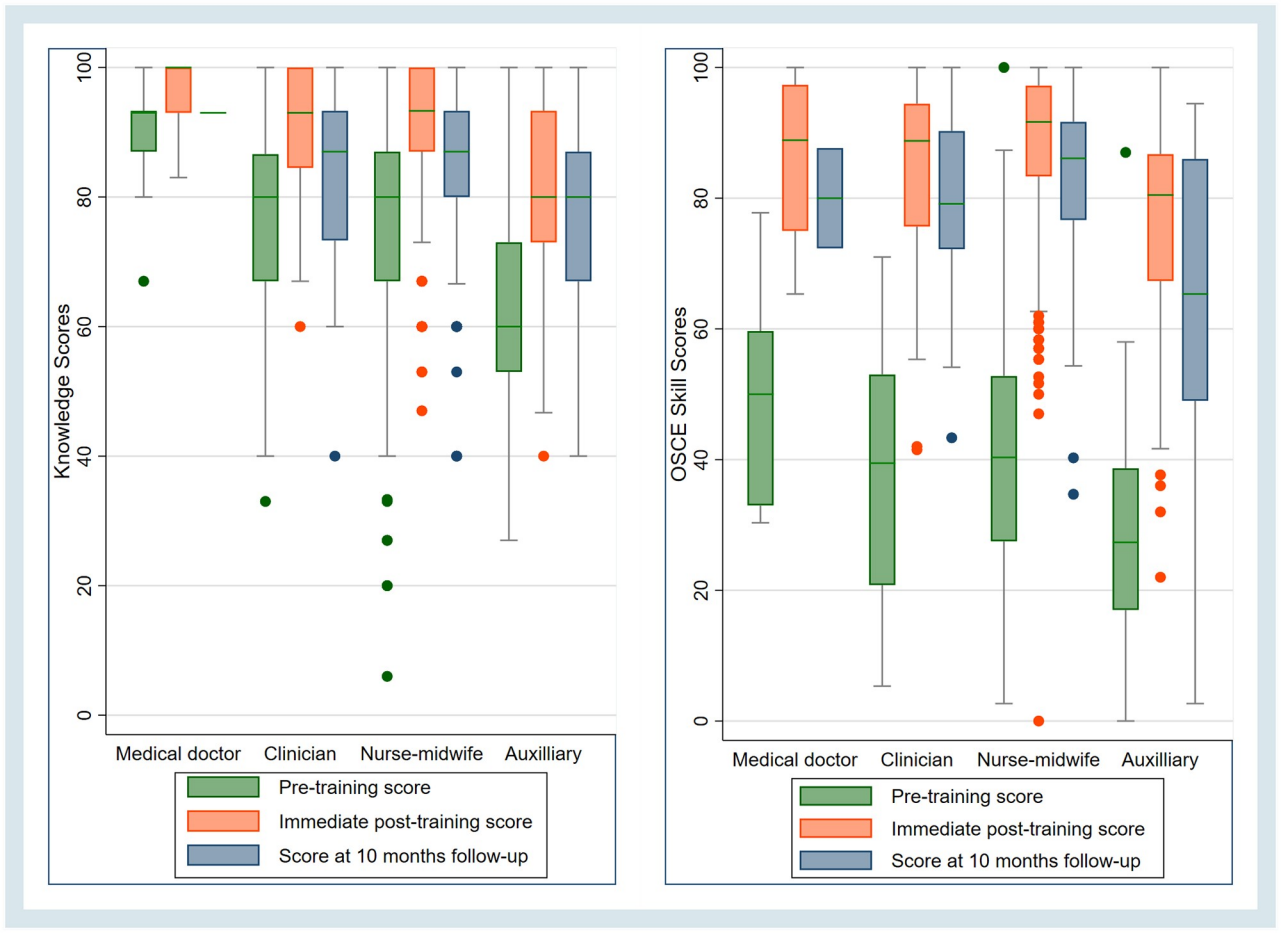
Box plot for knowledge (left) and OSCE skill score (right) on pre-training, immediately post training and 10-month follow-up for different health workers’ professions.

There was a significant overall decline of skills at 10-month follow-up from 85.4% (95% CI 84.3, 86.5) to 81.1% (95% CI 77.8, 88.2) with p-value 0.002. Skill decline was significant in auxiliary staff from 76.6% (95% CI 73.9, 79.3) to 63.8% (95% CI 54.7, 72.9, p-value < 0.000). Distribution of knowledge and skills scores at the different times per profession is shown in Fig 2.

In Table 3, we show the linear regression for mean skill score changes adjusted for pre-training scores and for immediate post-training scores with regression coefficient and 95% confidence interval. A similar table with mean knowledge score changes are attached as S1 Table. We observed an increase of 47.8 percentage-points (95% CI 46.5, 49.2, p-value <0.001) in skill score immediately post-training (Table 3). The 10-month follow-up skill score was -4.0% (95% CI -6.8, -1.2, p-value <0.001). Compared to nurse-midwives, auxiliary staff gained 7.6 percentage-points (p-value <0.000) less on skills immediately post-training, while medical doctors and other clinicians gained -3.4 and -2.7 percentage-points respectively (p-value >0.05) compared to nurse-midwives. Compared to health workers who assisted less than five deliveries in the last month, those assisting 6–15 deliveries gained 2.4 percentage-points more (95% CI 0.4, 4.5, p-value 0.021).

**Table 3:** Linear regression for mean skill changes immediately post-training and at 10-month follow-up of HMS BAB training.

*Health worker characteristic*	*n*	*Mean Skill Change of score between pre and post-training*	*Regression Coefficient adjusted for pre-training scores (95% CI)*	*n*	*Mean Skill Change of scores between 10-month follow-up and post-training*	*Regression Coefficient adjusted for post-training scores (95% CI)*
***All***	***552***	47.8 (46.5, 49.2)	-0.7 (-0.7, -0.6) ***	172	-4.0 (-6.8, -1.2)	-0.7 (-0.9, -0.5) ***
***Profession***						
*Medical doctors*	*6*	32.3 (24.6, 40.1)	-3.4 (-12.3, 5.5)	2	-9.9 (-20.7, 1.0)	-4.6 (-25.6,16.5)
*Other clinicians*	*46*	47.3 (43.4,51.2)	-2.67 (-6.0, 0.7)	16	-5.4 (-15.0, 4.2)	-5.6 (-13.5, 2.2)
*Nurse-midwives*	*382*	47.3 (45.8,49.1)	Reference	127	-2.1 (-5.1, 0.8)	Reference
*Auxiliary staff*	*115*	49.9 (47.1, 52.8)	-7.6 (-10.1, -5.2) ***	26	-11.4 (-21.2, -1.6)	-18.9 (-24.5, -12.2) ***
***Number of deliveries assisted in the last month***						
*≤ 5 del/month*	*288*	47.9 (46.1, 49.8)	Reference	87	-6.6 (-10.9, -2.2)	Reference
*6–15 del/month*	*196*	47.8 (45.5,49.9)	2.4 (0.4, 4.5) *	57	-2.2 (-6.2, 1.9)	6.9 (1.5, 12.2) *
*≥16 del/month*	*68*	47.3 (43.6, 51.1)	2.5 (-0.5, 5.5)	28	0.1 (-6.8, 67.0)	7.0 (0.2, 13.8) *
***Years of professional experience***						
*< 2 years*	*144*	50.1 (47.5, 52.8)	Reference	47	-3.52 (-8.5,1.5)	Reference
*2–4 years*	*172*	48.4 (46.1, 50.7)	1.4 (-1.0, 3.9)	50	-4.5 (-8.7, -0.3)	0.8 (-5.5, 7.1)
*5–7 years*	*68*	46.5 (42.6, 50.0)	-1.6 (-4.8, 1.5)	22	-9.3 (-19.4, 0.8)	-5.9(-13.9, 2.0)
*≥ 8 years*	*143*	46.7 (44.2,49.3)	-2.7 (-5.2, -0.2) *	40	-4.8 (-9.9, 0.3)	-2.1 (-8.8, 4.5)
***Facility level***						
*Hospital*	*275*	48.1 (46.2,49.9)	Reference	82	-2. 7(-6.8, 1.5)	Reference
*Health centres performing C- sections*	*66*	48.3(44.4,52.2)	1.8 (-2.9, 3.2)	30	-3.7 (-10.3, 2.3)	2.1 (-4.9, 8.9)
*Health centre*	*211*	47.3 (45.1, 49.5)	0.6 (-1.5, 2.6)	60	-6.1 (-10.9, -1.3)	-0.8 (-6.2, 4.7)
***Ever attended prior in-service AMTSL training***						
*Yes*	*271*	47.8 (45.9, 49.6)	Reference	72	-3.2 (-6.5, -0.01)	Reference
*No*	*266*	48.6 (46.7, 50.6)	-0.6 (-2.5,1.3)	90	-4.9 (-9.4, -0.37)	-2.9 (-7.9, 2.2)
***Ever attended prior in-service PPH training***						
*Yes*	*239*	47.6 (45.6,49.6)	Reference	68	-3.9 (-6.7, -1.1)	Reference
*No*	*283*	48.7 (46.8,50.6)	-2.6 (-4.5, -0.6) **	88	-3.9 (-8.5, 0.6)	-3.1 (-8.1, 1.8)

*p-value <0.05,

**p-value <0.01

***p-value <0.001

At follow-up we observed less skill decline among health workers with 6–15 and with >16 deliveries compared to the referent group, this was statistically significant (p-value 0.0012 and 0.043 respectively).

## Discussions

### Main findings

Our study indicated significant increase of health workers knowledge from 74.2% (95% CI 72.8, 75.5) to 89.2% (95% CI 87.5, 90.9) and skills from 38.2% (95% CI 36.8, 39.60) to 85.4% (95% CI 84.3, 86.5), a 47.8 percentage-point increase (95% CI 46.5, 49.2) immediately-after the training. Pre-training skill scores were lower than knowledge scores. Auxiliary staff showed a larger increase in scores from the training (49-percentage-point, 95% CI 47.1, 52.8) compared to other professions, but the 10-month follow-up assessment also indicated that this group had the largest decline of -11.4% (95% CI 21.2, -1.6).

Profession, number of deliveries assisted in the last month and years of professional experience were associated with skill change immediately post-training. Health workers assisting 6–15 deliveries in the last month gained 2.4 percentage-points more than those assisting <5 deliveries (p-value 0.021) immediately post-training and displayed less skill decline at 10-month follow-up. Health workers with more than 8 years of profession experience significantly scored less than those with <2 years of experience. Working in health centres that performed C-section and ever attending prior in-service PPH training (but not AMTSL) were associated with higher gain in skill scores.

Our study supports the existing evidence that the HMS BAB one-day training with mandatory weekly practice sessions is beneficial to upgrade health workers’ knowledge and skills in prevention, assessment and basic management of PPH [15, 22, 23]. Like other studies the median gain in skills was higher than for knowledge [20], perhaps due to less chance to improve the already high knowledge scores.

We observed the largest gain in skills score among auxiliary staff. Although auxiliary staff is not officially counted as ‘skilled birth attendants’, they assist deliveries as part of their routine care in the studied facilities. A recent publication by Rao et al. (2018) from India proposed that lower cadre health professionals that are not trained for delivery care can be trained and mentored to perform routine delivery care and manage PPH to the same level as nurse-midwives [31]. While this is an encouraging finding, our study indicated lower pre-training knowledge and skills as well as lower retention of knowledge and skills over time compared with nursing and clinical professions. Thus, the employment of auxiliary staff for childbirth care needs to be considered with caution as larger efforts and investments are needed to support them to maintain a minimum knowledge and skills level. At the same time, it is important to recognise that auxiliary cadres provide extra hands on the ground in a resource constrained setting with a limited workforce. Only 12–27% of auxiliary staff had ever attended in-service training in this study, as in Tanzania they are usually excluded from these training as they are not considered skilled. We believe they should be provided with such trainings so that they also know what needs to be done in case of emergencies.

Our study concur with similar studies of in-service trainings that reported being active in clinical duties in the maternity ward was associated with better skills and also more retention of acquired skills following a training [12, 19, 22]. To date there is no consensus as to what an acceptable number of deliveries in a month is that a health worker can manage, strengthen and maintain the learned skills without burn-out. In this study, health workers who self-reported to have ever attended a prior PPH in-service training had higher scores than those not attended. This was not observed for AMTSL in-service training. We could not explain this, however, we did not assess contributing factors like time that elapsed from the reported training or training modality. Such factors need to be explored in future studies.

Studies on knowledge and skills retention reported different results on retention depending on time from initial in-service training from 3–24 months [19, 20, 32]. Retention of skills was reported to be better in settings where there are regular local mentoring programs, refresher trainings or repeated assessments [20, 33, 34] and continuous skills drills like the ‘low dose high frequency’ approach used in HMS BAB [15, 20, 32]. The HMS BAB training emphasized simulated practice, repetition and discussions and was specifically developed to focus on more practical training [35]. In this study, all health facilities had a mandatory weekly scenario-based practice session for 8 weeks following the initial training, led by local peers that could have led to the limited observed decline in knowledge and skills scores. Other factors that have been found associated with a skill decline in similar training modules were lower level facilities [20], prior in-service training and years of professional experience [35].

We were only able to include 63.7% of health workers in our 10-month assessment due to a variety of reasons illustrated in Fig 1. Only 9.5% of staff were transferred out of maternity wards to other departments which is an encouraging finding. High turnover and rotation of health workers from maternity wards is a common practice in the country, however not much has been documented about its potential for negative impact on individual health workers’ knowledge and skill retention and practice, team response when managing complications such as PPH and maternal health outcomes.

### Methodological consideration

The strength of our study is the large number of participants which allowed us to disaggregate the effects of the training in several sub-groups. We included 636 participants from 61 facilities: 605 had complete immediate post/pre-training knowledge scores and 591 complete immediate post/pre-training skills scores. Previous studies had smaller sample size of between 30 to 145 health workers [22, 24].

Another strength is that we used OSCE assessment for observation of simulated skills and used the same assessment tools during all the assessments periods, hence improve comparability. We report on aggregate skill score and change for three core competences at 10-month follow-up: rather than single skill scores as we were interested in a single score that may represent prevention and basic management of PPH portraying a more comprehensive score.

However, our study has limitations. We chose the 10-month follow-up for reassessing knowledge and skills retention, for pragmatic reasons, as we provided at this time period the training to the comparison facilities after the finalisation of the HMS BAB trial. While other authors did follow-up at several points, this was financially not possible for this study. Another limitation is that we missed fourteen health workers due to poor road infrastructures. We only reassessed 63.7% of the eligible number of participants, the remainder were absent or refused. We do not know whether those who refused or could not participate for other reasons had poorer or better performance and how this would have affected the results. However, the absentees were mainly medical doctors (3), other clinicians (14) and nurse-midwives (68), we hypothesize that perhaps their absence should not have affected overall results. Furthermore, our sample had few medical doctors at the initial training. We were only able to include two doctors and eleven clinicians in the 10-month assessment. Seven of thirteen medical doctors included in the training were interns and had moved to other employment areas. This limits the generalisability of our findings in relation to clinical professions. We also acknowledge that using same assessment tools for before and after assessment could mislead as health workers may be familiar with the questions and could have studied them to pass.

As part of the overall HMS BAB trial, health workers were to perform weekly practice drills for 8 weeks. We assumed all those trained in January 2016 had an opportunity to have done the practice drills as outlined by the intervention prior to the 10-month follow-up however, this may not be true. Twenty-three of 31 facilities trained in January 2016 completed the weekly practice drills within three months, three facilities did five sessions and five facilities did two sessions at this time. Although there were logbooks that showed participation of health workers during weekly drills, it was not possible to link individual providers to the number of drills attended and the decline of knowledge and skills. Nonetheless, a recent publication by William et al (2019) utilizing similar training practice drills reported less than one practice session per 8-weeks per health worker [36].

We report here on test scores. While preferably we would have reported on improved clinical practice, such assessments are logistically and practically difficult. However, we believe that our findings reflect training outcomes of improved test scores and retention was also reflected in the findings of the larger HMS BAB trial [13].

For some categories, for example the number of deliveries assisted in the last month or years of professional experience We have used cut-off points informed by mean and median exploration of the data for some categories. There are no clear standards and different authors have used different cut-off points which reduces comparability [12, 35, 37]. This may differ depending on the context, but there need to be some framework to inform on acceptable number of deliveries assisted in the last month per health worker [38].

### Implication for practice

Our study illustrates the potential of the HMS BAB training to improve knowledge and skills of health workers in maternity wards. While all health professionals benefited from the training, auxiliary staff benefited most, but they also had the lowest skills retention. This finding cautions the integration of lower trained staff into service delivery and a careful context specific assessment of advantages and disadvantages needs to be done if such professionals are used to fill shortages of qualified staff.

In-service training is widely used to improve health worker performance and the relevance has been underscored by systematic reviews [8, 9]. The HMS BAB training, in contrast to other trainings which are offered outside the facility premises, includes all health workers regardless of their professional background. We believe this is a strength of the training and our results underline the relevance for all professions. However, our study provides information on the difficulties to train all maternity health workers and to create stable teams. While twenty-five health workers dropped out early, others did not finish all the training components of the training as they were called to attend patients. About one-third of trained health workers were absent at 10-month follow-up because of various reasons. Training and other quality improvement initiatives need to be sensitive to such attrition and one-off interventions are likely to be insufficient.

Our study highlights gaps around understanding health workers characteristics that can strengthen training and use of resource.

## Conclusion

The HMS BAB was effective to improve knowledge and skills of health workers in the included hospitals and health centres. Both knowledge and skills increased significantly following the one-day HMS BAB training, followed by weekly practice drills for 6–8 weeks. Retention at 10-month follow-up was high. Profession and number of deliveries in the last month were associated with better skill outcomes and less decline at 10-month follow-up. We believe that more research on knowledge and skills decline need is needed to help facility management and policy makers plan for sustaining skills and performance of available health workers.

## Supporting information

S1 Fig Numbers of health workers for assessments at pre, immediately post-training and 10-month follow-up. (TIF)

S1 Table Linear regression of mean scores for knowledge change immediately after the training and at 10 months follow-up adjusted for pre- and immediate post-training score. (DOCX)

S1 Checklist STROBE statement—Checklist of icems that should be included in reports of *cross-sectional studies*. (DOC)

S1 Appendix Knowledge questionnaire. (PDF)

S2 Appendix OSCE checklist. (PDF)

S1 Data Anonymized dataset. (DTA)
