# Supplementary figures and images for: Predictors of change of health workers’ knowledge and skills after the Helping Mothers Survive Bleeding after Birth (HMS BAB) in-facility training in Tanzania
Source: PLoS One. 2020 May 18;15(5):e0232983. doi: 10.1371/journal.pone.0232983 (PMC7234376; doi:10.1371/journal.pone.0232983)

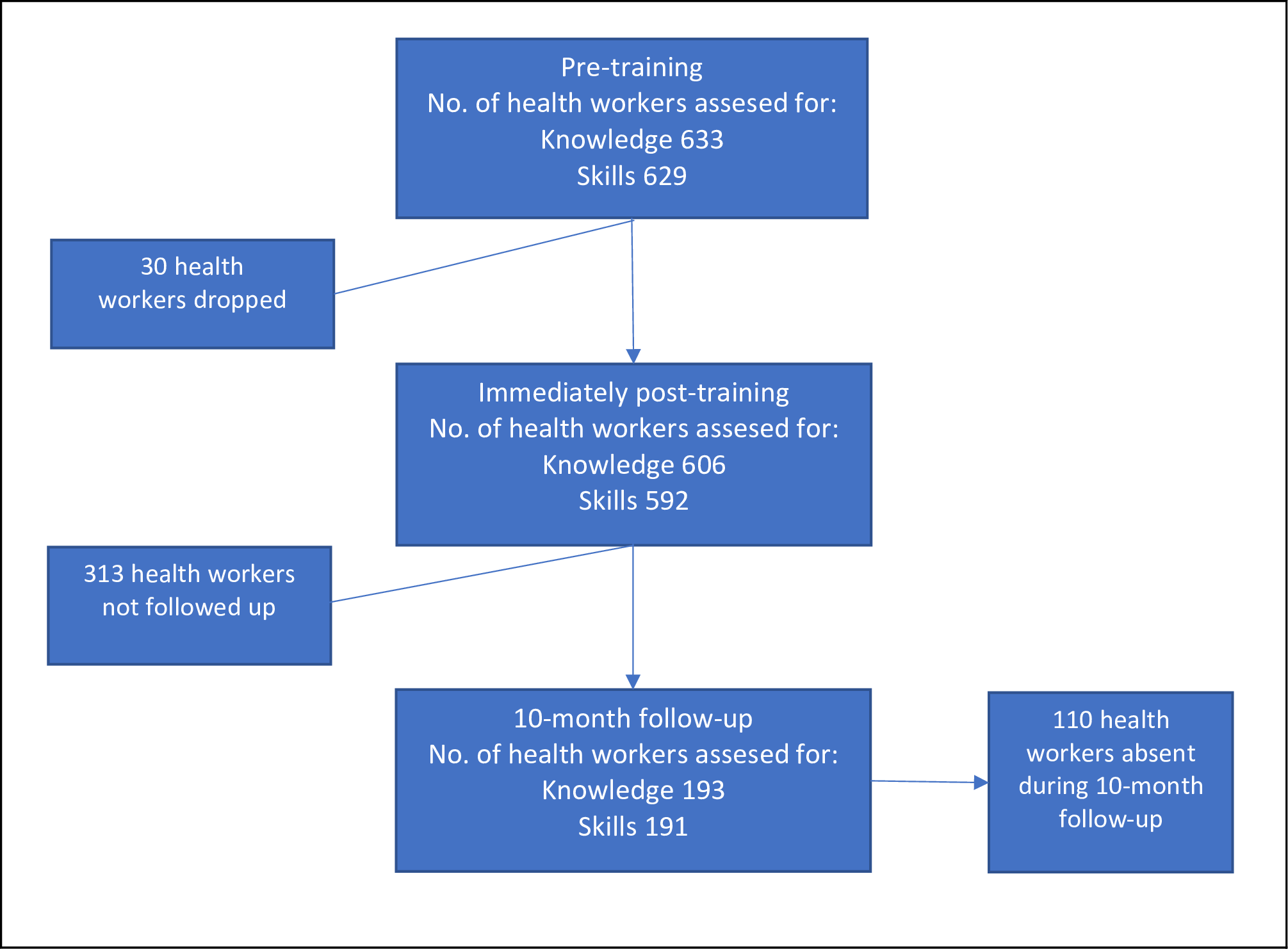

Supplement: S1 Fig — (TIF) [file pone.0232983.s001.tif]
