# Supplementary material for: Predictors of change of health workers’ knowledge and skills after the Helping Mothers Survive Bleeding after Birth (HMS BAB) in-facility training in Tanzania
Source: PLoS One. 2020 May 18;15(5):e0232983. doi: 10.1371/journal.pone.0232983 (PMC7234376; doi:10.1371/journal.pone.0232983)
Supplement: S1 Table — (DOCX) [file pone.0232983.s002.docx]

**Supplementary table. Linear regression of mean scores for knowledge change immediately after the training and at 10 months follow-up adjusted for pre- and immediate post-training score**

| ***Health worker characteristic*** | ***N*** | ***Mean change in knowledge (after-before)*** | ***Regression adjusted for pre-training scores*** | ***N*** | ***Mean change in knowledge (10 months follow-up-after training)*** | ***Regression co-efficient adjusted for after training scores*** |
| --- | --- | --- | --- | --- | --- | --- |
| ***All*** | 603 | 15.3 (14.3-16.3) | -0.5 (-0.5, -0.4) *** | 181 | -3.9 (-6.3, -1.5) | -0.7 (-0.9, -0.5) ** |
| ***Profession*** | | | | | | |
| *Medical doctors* | *11* | 4.49 (0.95, 8.02) | -2.8 (-8.3, 2.6) | 2 | 1.5 (-15.3,18.3) | 5.9 (-13.2, 25.2) |
| *Clinicians* | *51* | 14.4 (10.7, 18.0) | -1.0 (-3.6, 1.7) | 15 | -3.9 (-13.9,6.1) | -0.8 (-8.1, .) |
| *Nurse-midwives* | *414* | 14.5 (13.3, 15.6) | Reference | 135 | -4.5 (-6.9, -2.1) | Reference |
| *Auxilliary staff* | *123* | 19.4 (16.9,21.9) | -4.1 (-6.1, -2.1) *** | 29 | -0.8 (09.1, 7.4) | -9.2 (-15.4, -2.9) ** |
| ***Number of deliveries assisted in the last month*** | | | | | | |
| *≤ 5 del/month* | *315* | 15.3 (13.9, 16.8) | Reference | 91 | -4.5 (-7.9, -1.2) | Reference |
| *6-15 del/month* | *204* | 15.4 (13.7, 17.2) | 1.2 (-0.4, 2.8) | 60 | -5.0 (-8.9, -1.1) | 1.2 (-3.4,5.8) |
| *≥16 del/month* | *84* | 14.6 912.2.17.0) | -0.3 (-2.6, 1.9) | 31 | -.0 (-6.6, 6.6) | 4.3 (-1.4, 10.0) |
| ***Years of professional experience*** | | | | | | |
| *< 2 years* | *161* | 16.1 (14.1, 18.2) | Reference | 51 | -3.7(-8.6, 1.1) | Reference) |
| *2-4 years* | *183* | 14.3 (12.6, 16.1) | -0.5 (- 2.4. 1.5) | 51 | -5.2 (-9.7, -0.7) | 0.8 (-4.7, 6.3) |
| *5-7 years* | *71* | 13.7 910.8, 16.6) | -1.0 (-3.1, 1.5) | 24 | -3.1 (-11.1, 4.8) | 1.7 (-5.2, 8.5) |
| *≥ 8 years* | *153* | 16.1 (14.1, 18.0) | -0.1 (-2.2, 1.9) | 42 | -2.9 (-6.3, 0.5) | 2.4 (-3.3, 8.2) |
| ***Facility level*** | | | | | | |
| *Hospital* | *314* | 15.2 (13.8,16.6) | Reference | 91 | -4.7 (-8.0, -1.4) | Reference |
| *Health centres performing C- sections* | *66* | 14.3 (11.8, 16.7) | -3.1 (-5.6, -0.7) * | 31 | 1.0 (-5.1, 7.1) | 4.0 (-1.6, 9.7) |
| *Health centre* | *223* | 15.7 (14.0, 17.4) | -0.9 (-2.4, 0.7) | 60 | -5.2 (-9.4, -1.1) | -0.5 (-5.0, 4.1) |
| ***Ever attended prior in-service AMTSL training*** | | | | | | |
| *Yes* | *290* | 14.0 (12.6, 15.3) | Reference | 76 | -3.9 9-7.5, -0.3) | Reference |
| *No* | *290* | 16.0 (14.6, 17.5) | -0.1 (-1.5, 1.5) | 95 | -3.2 (-6.5, 0.1) | -0.8 (-5.0, -0.5) |
| ***Ever attended prior in-service PPH training*** | | | | | | |
| *Yes* | *255* | 13.1 (11.7, 14.4) | Reference | 71 | -4.7 9-8.8, -0.6) | Reference |
| *No* | *309* | 16.9 (15.4, 18.4) | 0 (-1.5, 1.6) | 94 | -2.6 (-5.6, 0.4) | 0.6 (-3.7, 4.9) |
