# Supplementary material for: Predictors of change of health workers’ knowledge and skills after the Helping Mothers Survive Bleeding after Birth (HMS BAB) in-facility training in Tanzania
Source: PLoS One. 2020 May 18;15(5):e0232983. doi: 10.1371/journal.pone.0232983 (PMC7234376; doi:10.1371/journal.pone.0232983)
Supplement: S2 Appendix — (PDF) [file pone.0232983.s005.pdf]

Helping Mothers Survive

Helping Mothers Survive: Bleeding After Birth
OSCE 1: AMTSL skills checklist

Guidelines to be read to participants:

- For each station assume you are alone in a rural health care facility with no surgical or blood transfusion capacity. You do have all equipment and supplies necessary for a normal vaginal birth.
- All essential information will be provided to you at the start of each OSCE station.
- Ask the evaluator to clarify any questions prior to beginning. Once the OSCE has started, the evaluator will not provide any further information.
- You will have 4 minutes to complete each station.
- Talk to and care for the woman in front of you exactly as you would in real life.
- Be explicit in verbalizing your clinical thinking and subsequent decisions.
- If you give a medication, you must state what you are giving, the dose, the route, and why you are giving it.

Key

Pass score for Routine Care 3rd stage = 9

Helping Mothers Survive: Bleeding after Birth
OSCE 1: AMTSL Skills Checklist

Participant name or ID# ____________________ Date ____________________

Routine care during the third stage of labor – Skill Check

Instructions for the Examiner

- **Briefly review the instructions for the participant.**
- **Start with baby on top of the simulator's abdomen.**
- **For cutting the cord, observe for: 1) changing/or taking off of first pair of gloves, 2) cutting after uterotonic.**
- **Observe only; do not intervene in demonstration of the participant.**
- **The feedback will be held at the end of the assessment for all learners.**

Read the following to the learner: "You are alone in a rural facility and at the start of this scenario you have just now delivered a baby and placed it on the mother's abdomen."

	**Checklist of skills**	**Yes** Performed to standard	**No** Did NOT perform to standard
		Check appropriate box for each item	
1.1	Following delivery of the infant, the provider checks for a second baby		
1.2	Tells the patient what medication she is being given		
1.3	Gives uterotonic medication within 1 minute of delivery of the infant		
1.4	Tells the patient why she is getting the medication		
1.5	Cuts the cord: observe for: 1) changing/or taking off of first pair of gloves, 2) cutting after uterotonic.		
1.6	Applies counter pressure when performing controlled cord traction		
1.7	Performs controlled cord traction only when the patient is having a contraction		
1.8	Uses both hands to catch the placenta		
1.9	Gently turns the placenta while it is being delivered		
1.10	Assesses fundal tone immediately following delivery of the placenta		
1.11	Inspects the placenta for completeness		
1.12	Checks the woman's bleeding		

Score __________/12 **Pass / Fail** (circle one)

OSCE 2: Retained Placenta checklist

Guidelines to be read to participants:

- For each station assume you are alone in a rural health care facility with no surgical or blood transfusion capacity. You do have all equipment and supplies necessary for a normal vaginal birth.
- All essential information will be provided to you at the start of each OSCE station.
- Ask the evaluator to clarify any questions prior to beginning. Once the OSCE has started, the evaluator will not provide any further information.
- You will have 4 minutes to complete each station.
- Talk to and care for the patient in front of you exactly as you would in real life.
- Be explicit in verbalizing your clinical thinking and subsequent decisions.
- If you give a medication, you must state what you are giving, the dose, the route, and why you are giving it.

Key

Pass score for OSCE 2 = 6

Helping Mothers Survive: Bleeding after Birth

OSCE 2: Retained Placenta Skills Checklist

Participant name or ID# ____________________ Date ____________________

Instructions for the Examiner

- **Briefly review the instructions for the participant.**
- **Keep the placenta attached in the simulator for the entire scenario. Do not release it during controlled cord traction.**
- **If the learner states the woman must be transported, ask, "What is to be done with the baby?"**
- **At the end of the scenario, if the learners states the woman must be transported, say, "Describe to me your transportation plan."**
- **Observe only; do not intervene in demonstration of the participant.**
- **The feedback will be held at the end of the assessment for all learners.**

Read the following to the learner: You are alone in a rural health facility. You gave a uterotonic medication within 1 minute of delivery, and have provided controlled cord traction during contractions and monitored your patient's bleeding for the past 30 minutes. She remains stable, but continues to bleed slowly, and her placenta has not delivered.

	**Checklist of skills**	**Yes**<br><br>**Performed to standard**	**No**<br>**Did NOT perform to standard**
		Check appropriate box for each item	
2.1	Repeats 10 IU IM oxytocin		
2.2	Provides controlled cord traction for each contraction		
2.3	Guards uterus while providing controlled cord traction		
2.4	Identifies that the placenta may be retained		
2.5	Identifies that the patient must be transported		
2.6	When the evaluator asks what is to be done with the baby, answers that the baby will be kept with the mother		
2.7	Communicates respectfully and provides needed information to the mother and family throughout		
2.8	If the learner says the mother needs transfer, say, "Describe to me your transportation plan." Is plan appropriate to context?		

Score __________/ 8 **Pass / Fail** (circle one)

OSCE 3: Severe PPH

Guidelines to be read to participants:

- For each station assume you are alone in a rural health care facility with no surgical or blood transfusion capacity. You do have all equipment and supplies necessary for a normal vaginal birth.
- All essential information will be provided to you at the start of each OSCE station.
- Ask the evaluator to clarify any questions prior to beginning. Once the OSCE has started, the evaluator will not provide any further information.
- You will have 4 minutes to complete each station.
- Talk to and care for the patient in front of you exactly as you would in real life.
- Be explicit in verbalizing your clinical thinking and subsequent decisions.
- If you give a medication, you must state what you are giving, the dose, the route, and why you are giving it.

Key
Pass score for OSCE 3 = 8

OSCE 3: Severe PPH Skills Checklist

Participant name or ID# ____________________ Date ____________________

Instructions for the Examiner

- **Briefly review the instructions for the participant.**
- **Have the placenta in a basin by your side and pull the cervical ribbon on the simulator closed prior to the scenario.**
- **Start with a soft uterus which never contracts. Have bleeding start out moderate, then increase.**
- **If the learner says the woman must be transported, ask, "Why can't I stay here, my bleeding is better?"**
- **Observe only; do not intervene in demonstration of the participant.**
- **The feedback will be held at the end of the assessment for all learners.**

Read the following to the learner: You are alone in a rural facility. You have given 10 units of oxytocin IM and performed controlled cord traction with 3 contractions resulting in delivery of the placenta.

	**Checklist of skills**	**Yes**<br>**Performed to standard**	**No**<br>**Did NOT perform to standard**
		Check appropriate box for each	
3.1	Massages the uterus		
3.2	Checks the woman's bleeding		
3.3	Inspects the placenta for any missing pieces		
3.4	Re-checks the uterus and bleeding		
3.5	Gives a second dose of medication telling what dose, route and why		
3.6	Re-checks bleeding and tone		
3.7	Puts on long gloves		
3.8	Explains to woman that he/she will be providing bi-manual compression		
3.9	Provides bi-manual compression		
3.10	Makes the decision to transfer		
3.11	Explains to the patient that they will need to be transported for advanced care		
3.12	Answers patient's question, "Why can't I stay here, my bleeding is better?" correctly. (Because she is at risk for complications that cannot be treated at this local facility, or is "too high risk," or "might bleed again," or "we don't have blood here".)		

Score __________/12 **Pass / Fail** (circle one)
